# Effects of anti-inflammatory [1, 2, 4]triazolo[4, 3-*a*] [1, 8]naphthyridine derivatives on human stimulated PMN and endothelial cells: an *in vitro *study

**DOI:** 10.1186/1476-9255-3-4

**Published:** 2006-03-28

**Authors:** Chiara Dianzani, Massimo Collino, Margherita Gallicchio, Mario Di Braccio, Giorgio Roma, Roberto Fantozzi

**Affiliations:** 1 Department of Anatomy, Pharmacology and Forensic Medicine, University of Turin, V P Giuria 9, 10125 Torino, Italy; 2 Department of Pharmaceutical Sciences, University of Genoa, Viale Benedetto XV 3, 16132 Genoa, Italy

## Abstract

**Background:**

[1,2,4] triazolo [4, 3-*a*][1,8]naphthyridine derivatives (including NF161 and NF177) were tested for anti-inflammatory, analgesic and antipyretic properties and for their effects on spontaneous locomotor activity in mice and acute gastrolesivity in rats. Both NF161 and NF177 appeared to be anti-inflammatory and analgesic agents without toxic effects or acute gastrolesivity, but NF161 showed stronger anti-inflammatory activity, whereas NF177 was more active as analgesic.

**Methods:**

An EIA kit was used to investigate the ability of NF161 and NF177 to affect prostaglandin E_2 _(PGE_2_) and prostacyclin (PGI_2_) production by human umbilical vascular endothelial cells (HUVEC).

The compounds' effects on the production of reactive oxygen species (ROS) by human polymorphonuclear cells (PMNs) were studied in an *in vitro *cell model, evaluating inhibition of superoxide anion (O_2_^-.^) production induced by *N*-formylmethionyl-leucyl-phenylalanine (FMLP). Their effects on PMN adhesion to HUVEC were also investigated; they were incubated with PMNs and endothelial cells (EC) and challenged by stimuli including Platelet Activating Factor (PAF), FMLP, Phorbol Myristate Acetate (PMA), Tumor Necrosis Factor-α (TNF-α) and Interleukin-1β (IL-1β). Adhesion was quantitated by computerized micro-imaging fluorescence analysis.

**Results:**

Neither compounds modified PGE_2 _or PGI_2 _production induced by IL-1α.

O_2_^-. ^production and myeloperoxidase release from PMNs stimulated by FMLP was inhibited in a dose- but not time-dependent manner by both [1,8]naphthyridine derivatives, NF161 being statistically more active than NF177 (P < 0.01).

The compounds inhibited adhesion evoked by the pro-inflammatory stimuli PAF, FMLP, TNF-α and IL-1β in a concentration-dependent manner in the 10^-6^–10^-4^M range, being more active when PAF was used as stimulus and inactive when cells were challenged by PMA. Both compounds acted both on PMN and HUVEC.

**Conclusion:**

Considering the interesting anti-inflammatory effects of these compounds in *in vivo *models and the absence of acute gastrolesivity, the study improved knowledge of anti-inflammatory properties of NF161 and NF177, also demonstrating their potential *in vitro*, through inhibition of O_2_^-. ^production, myeloperoxidase release and PMN adhesion to HUVEC. Negative results on PG production suggest a cyclooxygenase (COX)-independent mechanism.

## Background

There is currently considerable therapeutic interest in novel anti-inflammatory drugs with mechanisms other than the inhibition of cyclooxigenase activity, typical of nonsteroidal anti-inflammatory drugs (NSAIDs), and thus devoid of irritant effects on the gastric mucosa and suitable for use in chronic inflammatory diseases. COX is the enzyme that catalyses the first two steps in the biosynthesis of prostaglandins (PGs) from arachidonic acid [1]. About a decade ago it was demonstrated that COX exists as at least two distinct isoforms, COX-1 and COX-2, both responsible for PG production in a range of tissues. HUVEC are known to possess both COX isoforms. COX-1 is constitutive and its expression cannot be modulated, while COX-2 induction in HUVEC has been demonstrated in response to various proinflammatory cytokines, such as IL-1α and β and TNF-α [2,3].

**Figure 1:**
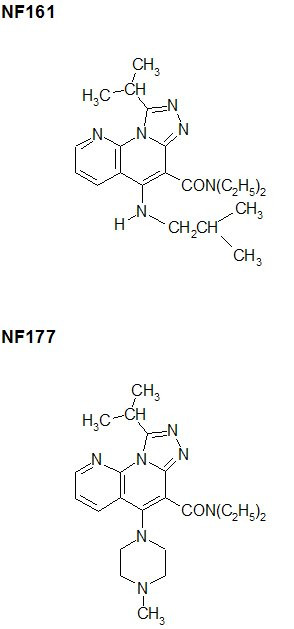
Structures of NF161 and NF177.

[1,8]naphthyridine derivatives have been reported to possess antibacterial [4], antymicobacterial [5], antitumoral [6], anti-inflammatory [7], antiplatelet [8], gastric antisecretary [9], antiallergic [10], local anaesthetic [11] and benzodiazepine receptor activities [12]. [1,8]naphthyridine derivatives are also reportedly associated with positive ionotropic [13], β-adrenergic blocking [14] and anti-hypertensive [15] activities. Recently, Roma et al. [16] synthesized a series of new derivatives, the substituted 5-amino[1,2,4]triazolo [4.3-*a*][1,8]naphtyridine-6-carboxamides, in order to obtain novel interesting anti-inflammatory agents. The authors showed that compound NF161 (Fig. 1) exhibited potent statistically-significant anti-inflammatory activity at the carrageenan-induced paw edema assay in rats, and showed interesting anti-aggressive activity (even if only at the highest dose) evaluated in the isolation-induced aggressiveness test in mice; results of a further test at 50 mg/Kg dose to evaluate antinociceptive activity of this compound were not statistically significant. This compound has since been demonstrated to have marked analgesic activity at the acetic-acid-induced writhing test in mice (at 200 and 100 mg/kg) and a complete lack of acute gastrolesivity in rats [17].

The same paper described a new compound (NF177, Fig. 1), belonging to the same chemical family [17]. In comparison with NF161, NF177 demonstrated stronger analgesic activity in the writhing test in mice, remarkably lower anti-inflammatory activity in the carrageenin-induced paw edema assay in rats (being active only at the highest dose), a marked effect on locomotor activity in mice and, like NF161, a complete lack of acute gastrolesivity in rats.

Bone-marrow-derived leukocytes, in particular PMNs, are responsible for much of the damage in chronic inflammation reactions. ECs play a crucial role in leukocyte homing to tissues, through adhesive interactions with these cells. These processes depend on specific cell adhesion molecule (CAM) activity in leukocytes and ECs. CAM activity can be regulated by modulating their expression or intrinsic adhesive functions [18]. PMNs maintain several of these receptors in an inactive state during transit in the bloodstream and extracellular fluids, and only activate them when the proper specific stimuli are delivered. An important role in this signaling is played by molecules belonging to the chemoattractant family, which comprises classic chemoattractants such leukotriene B_4_, PAF, *N*-formyl peptides and chemokines, which are cytokines with chemoattractant capacity and a high level of sequence homology. Chemoattractants bind to the surface of ECs, which present them to circulating PMNs. PMN interaction with chemoattractants promotes integrin adhesion via inside-out signaling [19], activates cell motility, and stimulates degranulation and respiratory burst of phagocytes [20].

Since PMN adhesion to ECs is a key step in the development and progression of inflammation, we investigated the anti-inflammatory activity of NF161 and NF177, evaluating their ability to inhibit PMN adhesion to HUVEC, induced by several stimuli; we also studied PMN adhesion to FCS-coated plastic wells. PMN activation was determined via O_2_^- ^production and myeloperoxidase release.

Both NF161 and NF177 inhibited O_2_^-. ^production and myeloperoxidase release from PMNs, challenged by FMLP, NF161 being statistically more active than NF177. Moreover, both compounds inhibited the PMN adhesion to HUVEC evoked by pro-inflammatory stimuli, acting either on PMNs or on HUVEC. Neither NF161 nor NF177 was able to modify the PGE_2 _and PGI_2 _production induced by IL-1α in HUVEC, thus suggesting a COX-independent mechanism of action.

## Methods

### Materials

Dextran T500 was from Pharmacia Biotech (Uppsala, Sweden). Bovine calf serum (BCS, endotoxin tested) was from Hyclone Laboratories Inc. (Logan, UT). Trypsin was from Difco Laboratories Inc, Detroit, MI. Histopaque^®^1077, fluorescein diacetate, M199 (endotoxin tested), PAF, *n*-FMLP, IL-1β, PMA, TNF-α, cytochrome C, superoxide dismutase, cytochalasin B, HAS, 2-chloroadenosine and methylthiazolyldiphenyl-tetrazolium bromide (MTT) were from Sigma-Aldrich (St. Louis, MO). Collagenase was from Roche Diagnostics (Mannheim, Germany). MAb LFA-1 (recognizes CD11a) was a gift from Prof. U. Dianzani (University of Eastern Piedmont, Novara, Italy). MAb OKM-1 (recognizes CD11b).

was obtained from the American Type Culture Collection (Rockville, MD, USA). Each mAb was used at the concentration that demonstrated maximal inhibitory effects in the adhesion assay, (20 μg/ml) [21].

All other reagents and solvents were from Merck (Darmstadt, Germany). The [1,8]naphthyridine derivatives were synthesized as reported in Grossi et., 2005 [17]. Each of the [1,8]naphthyridine derivatives was dissolved in dimethyl sulphoxide (DMSO). Stock solutions were prepared daily and diluted in M199 to the appropriate concentrations before each experiment. The final concentration of DMSO was never above 0.1%. The same amount of DMSO was added to controls and did not affect absolute control adhesion nor superoxide anion production (O_2_^-.^).

### Cell cultures

PMNs were prepared from citrated venous blood obtained from healthy volunteers at a local hospital bank, using the standard techniques of dextran sedimentation followed by Histopaque^®^1077 gradient centrifugation. Residual erythrocytes were removed by hypotonic lysis and PMNs were resuspended in buffered salt solution (BSS) (138 mmol/l NaCl, 2.7 mmol/l KCl, 8.1 mmol/l Na_2_HPO_4_, 1.5 mmol/l KH_2_PO_4_, 1 mmol/l MgCl_2_, 1 mmol/l CaCl_2_, pH 7.4) supplemented with 1 mg/ml glucose and 1 mg/ml human serum albumin (HSA). Purity of the final cell suspension and cell viability, assessed by the trypan blue exclusion test, were > 95% in all cases. Cell viability was not affected by compound treatments, measured with the MTT test [22] for HUVEC and with the trypan blue exclusion test for PMNs (test time: 0–25 min; concentration 10^-4^M).

HUVEC were isolated as described elsewhere [23] from human umbilical veins by collagenase treatment (0, 1%) and cultured on gelatin-coated culture dishes in M199 medium supplemented with 20% heat-inactivated bovine calf serum (BCS), 100 U/ml penicillin, 100 μg/ml streptomycin, 5 U.I./ml heparin, 12 μg/ml bovine brain extract and 200 mM glutamine. HUVEC were grown to confluence in flasks and used at the II-IV passages.

### Superoxide anion (O_2_^-.^) production

PMNs (1 × 10^-6^cells/ml) were suspended in buffer saline solution (BSS); they were pretreated with cytochalasin B (5 μg/ml) for 5 min at 37°C to maximize the measured response, then challenged with the test compound for 15–25 min at 37°C before exposure to 10^-7^M FMLP for a further 5 min. O_2_^-. ^production was determined spectrophotometrically by measuring the superoxide dismutase-inhibitable reduction of cytochrome C reduced/10^6 ^PMNs/min. NF161 and NF177 were checked for interference in the assay by measuring their effects on cytochrome C reduction with a xanthine oxidase superoxide generating system. Neither of the compounds tested interfered with the spectrophotometric assay. Assays were carried out in the same buffer, with 100 μM cytochrome C, 150 μM hypoxanthine, 0.01 units of xanthine oxidase per milliliter, and appropriate concentrations of each compounds.

### Myeloperoxidase assay

PMNs (2 × 10^6 ^cells) were incubated in BSS to a final volume of 1 ml with cytochalasin B and NF161 or NF177 for 15 min, than FMLP (10^-8^M) was added for a further 5 min. Myeloperoxidase release was assayed as described by Henson et al. [24]. Release of enzymes was expressed as percentage of total cell enzyme activity.

### Adhesion assay

HUVEC were grown to confluence in 24-well plates. PMNs (10^7^cells/ml) were labeled with fluorescein diacetate (5 μg/ml) for 30 min at 37°C, washed with BSS, and plated at 10^6 ^cells/well in a final volume of 0.25 ml BSS on HUVEC pretreated with the 1, 8-naphtyridines (10^-6^–10^-4^M) for 10 min and challenged with various stimuli: FMLP and PAF (both at 10^-7^M) or PMA (10^-8^M) for 10 min or IL-1β and TNF-α (both at 10 ng/ml) for 1 h. After incubation, non-adherent PMNs were removed by washing three times with 1 ml BSS. The center of each well was analyzed by fluorescence image analysis [25]; adherent cells were counted using Image Pro Plus Software for micro-imaging (Media Cybernetics, version 5.0). Single experimental points were assayed in quadruplicate, and the standard error of the four replicates was below 10% in all cases. Data are presented as percentage adhesion versus the control value, control adhesion being measured on HUVEC that underwent no treatment. Control adhesion was 51 ± 9 cells/microscope fields (n = 30).

The direct effect on HUVEC was assessed by pretreated the cells with the 1, 8-naphtyridines (10^-4^M) and 10^-7^M PAF for 20 min, washed three times and challenged with PMNs for further 10 min.

The direct effect on PMNs was assessed by seeding the cells on 24-well EC-free plates for 20 min at 37°C, in the presence of NF161 or NF177 and 10^-7^M FMLP. The plates had previously been coated with heat-inactivated calf serum for three hours to reduce spontaneous adhesion to the plastic wells. Percentage inhibition of adhesion was calculated as follows: [100 - (*a*)/(*b*)] × 100, where *a *is adhesion measured in the presence of the compound plus stimulus minus basal adhesion, and *b *is adhesion elicited by stimulus minus basal adhesion.

### Prostacyclin and Prostaglandin E_2 _enzyme immunoassay

The effect of IL-1α on the release of PGE_2 _and the stable metabolite of prostacyclin, 6-keto-PGF_1α_, were established by incubating HUVEC in the presence of IL-1α (10 ng/mL) for 20 h. After incubation, the medium was collected and analyzed for PGE_2 _and 6-keto-PGF_1α_. On the basis of the data obtained in this experiment, the effect of NF161 and NF177 on the release of PGE_2 _and 6-keto-PGF_1α_, in IL-1α-treated HUVEC was studied. HUVEC were pretreated for 20 h with IL-1α plus either NF177 or NF161 (10^-6^–10^-4^M). In a second set of experiments, HUVEC were pre-treated with IL-1α for 20 h and then with NF177 or NF161 (10^-6^–10^-4^M) for a further 30 min. The PGE_2 _and 6-keto-PGF_1α _content of the media were assayed using the Amersham Biosciences enzyme immunoassay kit (Amersham Biosciences Corp., Piscataway, NJ, USA) and following the manufacturer's protocol.

### Statistical analysis

**Figure 2:**
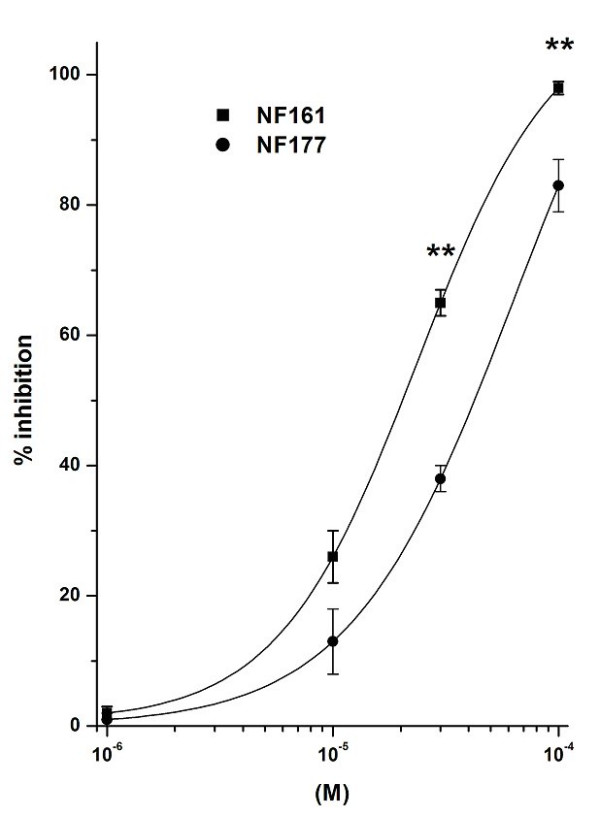
Effects of NF161 and NF177 on FMLP-evoked O_2_^-. ^production by PMNs. The results are expressed as percentage inhibition of O_2_^-. ^production evoked by 10^-7^M FMLP in the absence of NF161 and NF177: this production amounted to 2.1 ± 0.3 nmol cytochrome C reduced/10^6 ^cells/min and was taken as 100%. Data are expressed as means ± SEM; n = 5. Asterisks mark statistically significant inhibition of NF161 versus NF177 (**P < 0.01).

**Figure 3:**
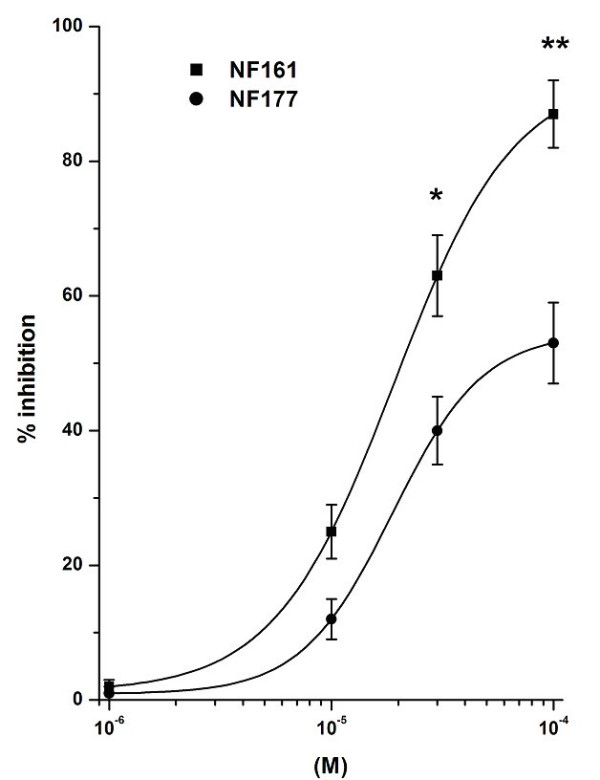
Effects of NF161 and NF177 on FMLP-evoked myeloperoxidase release by PMNs. The results are expressed as percentage inhibition of myeloperoxidase release evoked by 10^-8^M FMLP in the absence of NF161 and NF177: this release amounted to 31 ± 6%. Data are expressed as means ± SEM; n = 5. Asterisks mark statistically significant inhibition of NF161 versus NF177 (*P < 0.05, **P < 0.01).

**Figure 4:**
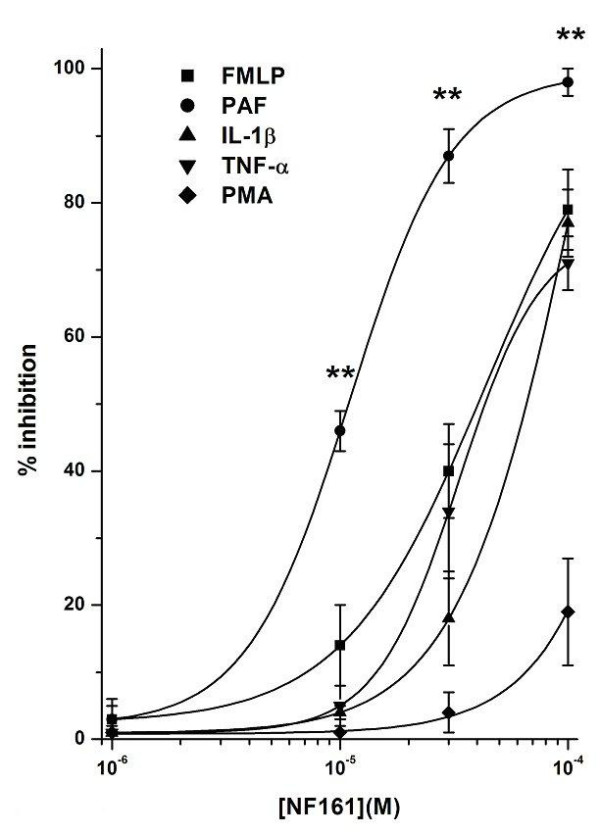
Effects of NF161 on PMN adhesion to HUVEC evoked by PAF, FMLP, IL-1β, TNF-α and PMA. HUVEC were pretreated with increasing concentrations (10^-6^–10^-4^M) of the tested compound for 10 min at 37°C and then challenged with PAF, FMLP (both at 10^-7^M) or PMA (10^-8^M) and PMNs for a further 10 min; IL-1β and TNF-α (both at 10 ng/ml) were incubated 1 h with the pretreated HUVEC and then challenged with PMNs for a further 10 min. Data are expressed as percentage inhibition versus control adhesion. Control adhesion was 51 ± 9 cells/microscope field (mean ± SEM, n = 26). PAF, FMLP, IL-1β, PMA and TNF-α stimulation vs control was, respectively: 305 ± 37%, 283 ± 28%, 270 ± 23%, 340 ± 46% and 304 ± 29%. Data are expressed as means ± SEM; n = 5. IC_50 _values were: 9.2 ± 0.7 × 10^-6^M, 3.7 ± 0.3 × 10^-5^M, 6.4 ± 0.4 × 10^-5^M and 3.1 ± 0.5 × 10^-5^M when the stimulus used was PAF, FMLP, IL-1β and TNF-α, respectively. IC_50 _values obtained with PAF were statistically different from all others (P < 0.01). Asterisks mark statistically significant inhibition of NF161 using PAF as stimulus versus all other stimuli (**P < 0.01).

**Figure 5:**
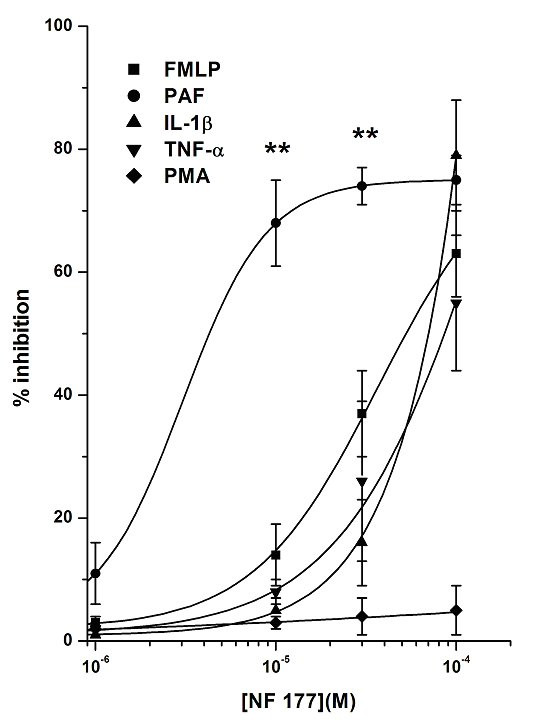
Effects of NF177 on PMN adhesion to HUVEC evoked by PAF, FMLP, IL-1β, TNF-α and PMA. HUVEC were pretreated with increasing concentrations (10^-6^–10^-4^M) of the tested compound for 10 min at 37°C and then challenged with PAF, FMLP (both at 10^-7^M) or PMA (10^-8^M) and PMNs for a further 10 min; IL-1β and TNF-α (both at 10 ng/ml) were incubated for 1 h with the pretreated HUVEC and then challenged with PMNs for a further 10 min. Data are expressed as percentage inhibition versus control adhesion. PAF, FMLP, IL-1β, PMA and TNF-α stimulation vs control was, respectively: 305 ± 37%, 283 ± 28%, 270 ± 23%, 340 ± 46% and 304 ± 29%. Data are expressed as means ± SEM; n = 5. IC_50 _values were: 1.7 ± 0.6 × 10^-6^M, 2.6 ± 0.5 × 10^-5^M, 9.4 ± 0.4 × 10^-5^M and 3.9 ± 0.5 × 10^-5^M when the stimulus used was PAF, FMLP, IL-1β and TNF-α, respectively. IC_50 _values obtained with PAF were statistically different from all others (P < 0.01). Asterisks mark statistically significant inhibition of NF177 using PAF as stimulus versus all other stimuli (**P < 0.01).

**Figure 6:**
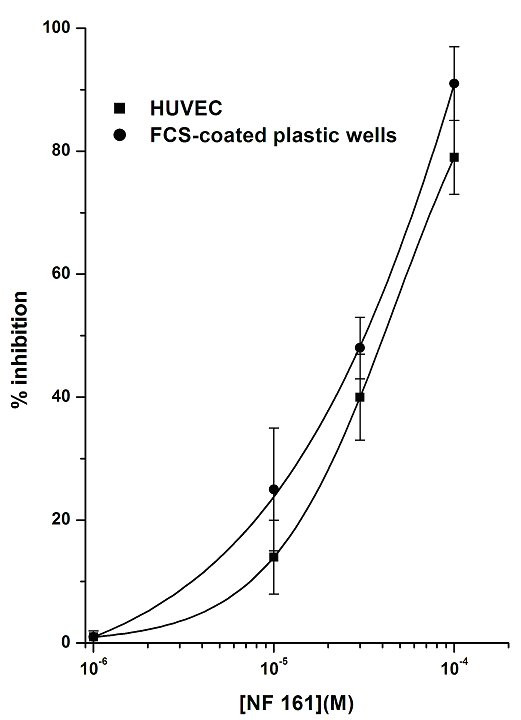
Effects of NF161 on PMN adhesion to FCS-coated plastic wells compared with PMN adhesion to HUVEC. PMNs were incubated with increasing concentrations (10^-6^–10^-4^M) of the tested compound for 10 min at 37°C and then challenged with FMLP 10^-7^M for 10 min. FMLP stimulation vs control was: 323 ± 33%. Data are expressed as percentage inhibition versus control adhesion, as means ± SEM; n = 5.

Results are expressed as means ± SEM; *n *indicates the number of experiments. Data in Fig. 2, 3, 4, 5, 6, 7 were analyzed by two-way analysis of variance to ascertain whether differences among the means were significant. The Bonferroni multiple comparison post-test was then applied to determine significant differences between specific pairs of means. The molar concentration of each compound that reduces response to the stimulus by 50% (IC_50_) was calculated with a non-linear regression model using the software Origin version 6.0 (Microcal Software, Northampton, USA). IC_50 _data in Figures 4 and 5 were analyzed using one-way analysis of variance, followed by the Tukey multiple comparison post-test. Differences were considered to be statistically significant at a value of P < 0.05. All statistical analyses were done using GradPadPrism 3.0 software (GraphPad Software, San Diego, California, USA).

**Figure 7:**
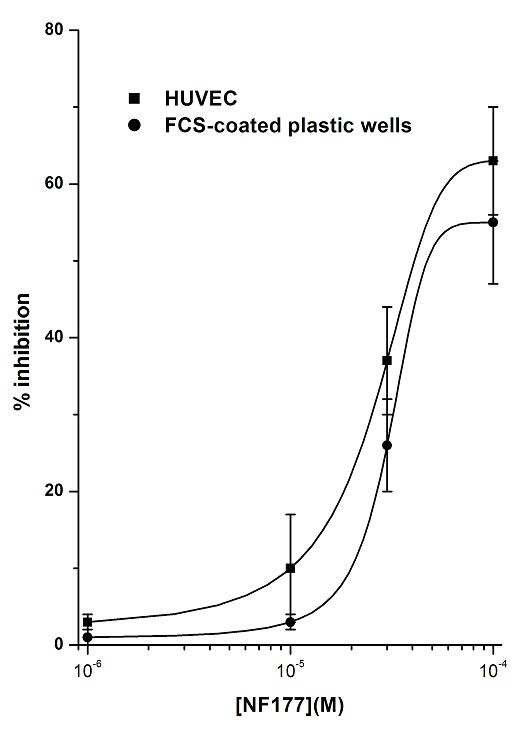
Effects of NF177 on PMN adhesion to FCS-coated plastic wells compared with PMN adhesion to HUVEC. PMNs were incubated with increasing concentrations (10^-6^–10^-4^M) of the tested compound for 10 min at 37°C and then challenged with FMLP 10^-7^M for 10 min. FMLP stimulation vs control was: 323 ± 33%. Data are expressed as percentage inhibition versus control adhesion, as means ± SEM; n = 5.

## Results and discussion

### Effect of NF161 and NF177 on IL-1α-induced PGE_2 _and PGI_2 _production by HUVEC

The compounds NF161 and NFF177 had previously been tested in vivo for their anti-inflammatory effects [16,17]. In the carrageenin induced rat paw edema test, compound NF161 exhibited statistically significant anti-inflammatory activity in the dosage range 6.25–200 mg/Kg, while compound NF177 only did so at 200 mg/Kg. However, their mechanism of action had not been demonstrated. The aim of the present study was to investigate how these compounds exert their anti-inflammatory effects. The first mechanism we focused on was inhibition of the COX pathway, as COX is one of the most important enzymes involved in development of the inflammatory process. Most anti-inflammatory drugs exert their effects by inhibiting this pathway. COX has been found in two isoforms, and COX-2 is an inducible form responsible for the production of large amounts of pro-inflammatory PGs at sites of inflammation. For this reason we measured PG production in HUVEC. Caughey et al. [2] demonstrated that pre-treatment with IL-1α induces COX-2 expression in HUVEC, with a corresponding increase in PG release. Here, we report that IL-1α stimulation of HUVEC for 20 h induced COX activity, generating high level of PGE_2 _and the stable metabolite of prostacyclin, 6-keto-PGF_1α _(Table 1). PGE_2 _synthesis was drastically increased by IL-1α from the basal level (45 ± 8 pg/well) to 280 ± 29 pg/well (n = 3). Similarly, a massive release of 6-keto-PGF_1α _was detected in IL-1α-treated HUVEC. When NF161 or NF177 were added simultaneously with IL-1α, neither compound significantly modified PGE_2 _or PGI_2 _production in the 10^-6^–10^-4^M concentration range. Indomethacin 10^-5^M, used as positive control, inhibited PGE_2 _and PGI_2 _production almost completely (data not shown). These results demonstrate that NF161 and NF177 do not affect COX activity in HUVEC. As they do not modify PGE_2 _or PGI_2 _production. we might conclude that the mechanism of action of NF161 and NF177 does not involve COX enzymes (Table 1).

**Table 1:** Effect of NF161 and NF177 on PGE_2 _and PGI_2 _production by Il-1α-treated HUVEC

**(a)**	**PGE**_2_**(pg/well)**	**6-keto-PGF_1α _(pg/well)**	**(b)**	**PGE_2 _(pg/well)**	**6-keto-PGF_1α _(pg/well)**
**Control**	45 ± 8	797 ± 23	**Control**	58 ± 18	888 ± 30
**IL-1α**	280 ± 29	3391 ± 98	**IL-1α **	275 ± 22	4055 ± 22
**NF161 10^-4^M**	295 ± 31	3508 ± 82	**NF161 10**^-4^**M**	288 ± 17	3990 ± 38
**NF161 10^-5^M**	285 ± 23	3330 ± 49	**NF161 10**^-5^**M**	277 ± 28	3885 ± 33
**NF161 10^-6^M**	288 ± 28	3452 ± 74	**NF161 10**^-6^**M**	280 ± 22	3905 ± 55
**NF177 10^-4^M**	260 ± 21	3660 ± 84	**NF177 10**^-4^**M**	268 ± 17	3875 ± 42
**NF177 10^-5^M**	277 ± 15	3280 ± 94	**NF177 10**^-5^**M**	256 ± 22	4005 ± 35
**NF177 10^-6^M**	300 ± 24	3220 ± 77	**NF177 10**^-6^**M**	292 ± 16	4055 ± 44

1. Effects on PGE_2 _and PGI_2 _production by simultaneous treatment with IL-1α (10 ng/ml) and NF161 or NF177. Twenty hours later, PGE_2 _and the stable metabolite of prostacyclin, 6-keto-PGF_1α_, were measured in the sample prepared from the medium by EIA as described in the Materials and Methods section (n = 3).

2. Effects of NF161 and NF177 on PGE_2 _and PGI_2 _production by pre-induced COX-2. IL-1α (10 ng/ml) was added and incubated for 20 h. The cells were washed and NF161 or NF177 were added for a further 30 min. After incubation, PGE_2 _and 6-keto-PGF_1α _were measured in the sample prepared from the medium (n = 3)

### Effect on O_2_^-. ^production induced by FMLP on PMNs

Considering that the effects of NF161 and NF177 do not involve the COX pathway, we next investigated whether these compounds could interfere with other cellular mechanism(s) involved in the inflammatory process. With the aim of evaluating the ability of NF161 and NF177 to interfere with the oxidative burst of human neutrophils, we measured O_2_^-. ^production by PMNs in the presence and in the absence of these compounds. Neither of the tested compounds alone induced O_2_^-. ^production by PMNs in the concentration range 10^-6^–10^-4^M (data not shown). PMNs challenged with 10^-7^M FMLP released O_2_^-.^(2.1 ± 0.3 nmol citochrome C reduced/10^6 ^cells/min; n = 5). This concentration was selected as suitable to produce optimal O_2_^-. ^generation by human PMNs [26]. When PMNs were incubated with increasing concentrations of the two compounds (10^-6^–10^-4^M) for 10 min and then challenged with 10^-7^M FMLP, an inhibitory effect on O_2_^-. ^production was detected (Fig. 2). Both compounds dose-dependently inhibited O_2_^-. ^production evoked by 10^-7^M FMLP. Maximal inhibition was obtained at 10^-4^M. NF161 percentage inhibition vs control was 98 ± 1% and its IC_50 _= 2.1 ± 0.4 × 10^-5^M; the inhibition of O_2_^-. ^production induced by NF177 was lower (**p < 0.01: NF161 vs NF177), being 83 ± 4%, and its IC_50 _= 2.5 ± 0.5 × 10^5 ^M. Even when in cubation time was prolonged (30 min; data not shown), the substances tested were still able to inhibit O_2_^-. ^production, with a dose-response curve quantitatively and qualitatively equal to those depicted above in Fig. 2. 2-chloroadenosine, a well known modulator of PMN functions, used as positive control [27], caused greater inhibition than the tested compounds, with maximum effect of 70 ± 4% inhibition, and IC_50 _= 87 ± 6 × 10^-9^M. Fiorucci et al. [28] have shown that 10^-4^M indomethacin, a classical anti-inflammatory drug, caused notable O_2_^-. ^production, and Zimmerli et al. [29] demonstrated that 10^-5^M indomethacin did not inhibit superoxide production induced by FMLP. Consequently, in this experimental model, NF161 and NF177 acted through a different mechanism than that of indomethacin.

Reactive Oxygen Species (ROS), such as O_2_^-.^, are produced in all aerobic organisms during respiration and exist in the cell in equilibrium with endogenous antioxidants (e.g. glutathione, vitamins A, C and E). Excess production of ROS alters cellular redox balance. ROS react with many macro molecules causing structural and functional modifications, cytotoxicity and mutagenic damage [30]. ROS exert genomic effects and modulate cell proliferation, by activating transcription factors such as AP-1, AP-2 and NF-kB [31]. Leukocytes (particularly PMNs and monocytes) and ECs provide a rich source of ROS, that can contribute to the development of degenerative pathologies (e.g. atherosclerosis, diabetes, Alzheimer's disease, arthritis, multiplesclerosis). ROS also play an important role in evolving organ injury (e.g. cerebral, cardiac, intestinal damage), which characterizes the pathophysiology of ìschemia-reperfusion (I/R) [32]. Therefore the ability of these [1,8] naphthyridine derivatives to inhibit O_2_^-. ^production demonstrates their role in modulating oxidative stress and suggests their potential use in various degenerative diseases.

### Effect on myeloperoxidase release induced by FMLP on PMNs

With the aim of evaluating whether NF161 and NF177 are only capable of interfering with the O_2_^-.^production by PMNs, myeloperoxidase release in the presence and in the absence of these compounds was measured. Neither of the compounds tested induced myeloperoxidase release from non-stimulated PMNs in the concentration range 10^-6^–10^-4^M (data not shown). PMNs challenged with 10^-8^M FMLP were able to release myeloperoxidase (31 ± 6%; n = 5) and this concentration was selected as suitable for producing optimal myeloperoxidase release by human PMNs. When PMNs were incubated with increasing concentrations of the two compounds (10^-6^–10^-4^M) for 15 min and then challenged with 10^-8^M FMLP, an inhibitory effect on myeloperoxidase release was detected (Fig. 3). Both compounds dose-dependently inhibited myeloperoxidase release evoked by 10^-8^M FMLP. As in the O_2_^-. ^production test, NF161 induced a stronger inhibitory effect than did NF177; maximal inhibition was obtained at 10^-4^M. NF161 percentage inhibition vs control was 87 ± 5% and its IC_50 _= 1.7 ± 0.4 × 10^-5^M; the inhibition of myeloperoxidase release induced by NF177 was lower (**p < 0.01; *p < 0.05: NF161 vs NF177), being 53 ± 6% and its IC_50 _= 1.7 ± 0.3 × 10^-5^M.

### Effect of NF161 and NF177 on PMN adhesion to HUVEC induced by different stimuli: FMLP, PAF, IL-1β, TNF-α and PMA

Adhesion and transendothelial migration of leukocytes into the surrounding tissues are crucial steps in inflammation, immunity and atherogenesis [33,34,18]. The present experiments were designed to ascertain whether NF161 and NF177 exert inhibitory effects on PMN adhesion to HUVEC.

Control adhesion was 51 ± 9 cells/microscope field (mean ± SEM, n = 30). Neither of the substances (at 10^-6^–10^-4^M) affected PMN adhesion of resting cells (data not shown), while indomethacin, (10^-4^M), in the same experimental model, behaved differently, markedly increasing PMN adhesion to HUVEC [28]. Thus HUVEC were pretreated for 10 min with the tested compounds (10^-6^–10^-4^M) and then stimulated with different stimuli:10^-7^M FMLP and PAF, 10^-8^M PMA, 10 ng/ml TNF-α and IL-1-β (see Methods). 10^-7^M FMLP-induced adhesion was 283 ± 28% vs control: it has been shown that the tested concentration produces near-maximal activation of PMNs [35,36,20]. Concentrations of the other stimuli were chosen on the basis of published reports and our previous experimental results, in order to achieve an adhesion close to that obtained with FMLP [20,37,38].

Considering that the synthetic peptide FMLP, mimetic of bacterial chemotaxins, only activates PMN adhesive machinery, this stimulus was selected to evaluate the effect of the compounds tested on PMNs. TNF-α and IL-1-β preferentially act on EC [39-41], whereas PAF and PMA activate both PMNs and HUVEC [20].

NF161 showed a dose-dependent inhibitory effect in the concentration range 10^-6^–10^-4^M (Fig. 4), its inhibitory effect decreasing in the following order, depending on the stimulus used: PAF > FMLP TNF-α > IL-1-β > PMA (**P < 0.01, PAF vs FMLP, TNF-α, IL-1-β and PMA). The maximum inhibition was in all cases achieved at 10^-4^M, being 98 ± 2% with PAF, around 70–80% with FMLP, IL-1-β and TNF-α and below 20% with PMA.

Similar results were obtained with NF177 (Fig. 5), reaching higher inhibition effects using PAF and IL-1-β as stimulus and no effect with PMA. This stimulus selectivity was particularly interesting, because all the stimuli except PMA are known to activate HUVEC or PMNs through membrane receptors, whereas PMA is a direct protein kinase C (PKC) activator [42,43]. In the same experimental condition, mAbs against the integrin adhesion molecules LFA-1 and OKM-1 [44], used as positive controls, exerted 92 ± 3% and 94 ± 5% inhibition, respectively. These findings may suggest a direct action of these compounds on membrane structures and/or on CAMs, rather than through inhibition of an intracellular PKC pathway.

The role of CAMs in modulating PMN adhesion to ECs is well known. The CAMs involved in leukocyte trafficking constitute excellent targets for pharmacological modulation of the cellular response in inflammation. Several mechanisms can modulate the function of inflammatory CAMs, including competitive blockade, altered expression on the cell surface and interference with receptor activation. Several groups of pharmaceutical agents in use clinically interfere with the function of CAMs either directly or indirectly. Many clinical trials of anti-adhesion therapies have used humanized antibodies, but low-molecular-weight compounds have several advantages over antibodies and are less likely to trigger adverse immune reactions [45]. Therefore further direct evidence linking compounds NF161 and NF177 to their potential ability to interact with CAMs would be very useful to clarify their mechanism of action.

### Effects of NF161 and NF177 on PMNs or HUVEC

To better understand the selective effects of these compounds on the two different cell populations, we firstly evaluated their effects on PMN adhesion to FCS-coated plastic wells, using FMLP as stimulus. Comparison of the results obtained in this experimental system with those obtained previously using EC showed that the concentration-response curves were similar, without any significant difference, for both compounds (Fig. 6 and Fig. 7). Indeed, the ability of NF161 and NF177 to inhibit PMN adhesion can be regarded as a direct effect on PMNs, HUVEC being absent in this experimental model. Secondarily, we pretreated PAF stimulated HUVEC with (10^-4^M) NF161 or NF177, washed three times and then added PMNs. Also in this different situation, NF161 and NF177 are able to inhibit PMN adhesion to stimulated HUVEC (% inhibition of NF161 = 81 ± 7; % inhibition of NF177 = 67 ± 6; n = 5), demonstrating that NF161 and NF177 can also act on EC. Moreover, when we used PAF, which acts on both cell populations, the inhibition of PMN adhesion to HUVEC evoked by NF161 and NF177 was significantly higher than in all other cases. The concentration-response curves shifted leftwards by one order of magnitude for NF161 (Fig. 4) and by two for NF177 (Fig. 5), demonstrating a synergistic effect of NF161 and NF177 when both cell populations are stimulated simultaneously.

Leukocyte extravasation is due to the cooperative activity of several molecules acting on both PMNs and HUVEC; the rapid exertion of inhibition of adhesion, and the lack of activity of the two compounds when the stimulus was PMA, appear to indicate that NF161 and NF177 induce a steric block of adhesion molecules activated by pro-adhesive stimuli on PMNs and HUVEC. It is noteworthy that synthetic compounds directed against CAMs might be therapeutically effective partly because they block intracellular signaling events that are crucial for numerous immune cell activities, such as motile responses, exocytosis, cytokine production and the respiratory burst [46].

## Conclusion

In summary, our *in vitro *results indicate that the *in vivo *anti-inflammatory properties of compounds NF161 and NF177 [16,17] do not involve the COX pathway, but may be due to their anti-adhesive effects and their inhibition of O_2_^-. ^production and of myeloperoxidase release, these properties being shown *in vitro *with steep concentration-response curves and at high concentrations. The two compounds exert their anti-adhesive effects very quickly, inhibiting PMNs adhesion to HUVEC within 10–20 min, acting directly either on HUVEC or on PMNs. Moreover, the anti-adhesive effect increases markedly when both cell populations are activated at the same time. The inhibitory effect detected in the presence of FMLP, IL-1β and TNF-α is also particularly interesting, since these experimental conditions may be regarded as mimetic of pathophysiological mechanisms of inflammatory diseases. The features of NF161 and NF177, together with the *in vivo *anti-inflammatory effects reported by other authors for [1,8]naphthyridine derivatives and their absence of gastrotoxicity, may be interesting for the design and development of innovative anti-inflammatory molecules in this structural field.

## Abbreviations

PGE_2_: prostaglandin E_2_; PGI_2_: prostacyclin I_2_; HUVEC: Human Umbilical Vein Endothelial Cell; PMN: Polymorphonuclear cell; O_2_^-. ^: superoxide anion; FMLP: *N-*formylmethionyl-leucyl-phenylalanine; PAF: Platelet Activating Factor; EC: Endothelial Cell; PMA: Phorbol Myristate Acetate; TNF-α : Tumor Necrosis Factor-α IL-1β : Interleukin-1β FCS: Fetal Calf Serum; COX: cyclooxygenase; NSAIDs: Nonsteroidal anti-inflammatory drugs; CAM: Cell Adhesion Molecules; BSS: Buffer Salt Solution; BCS: Bovine Calf Serum; ROS: Reactive Oxygen Species; PKC: Protein Kinase C.

## Competing interests

The author(s) declare that they have no competing interests.

## Authors' contributions

CD and MC participated in the study design, carried out the experiments and wrote the manuscript. MG performed the ELISA experiments. GR and MD designed and synthesized NF161 and NF177. RF conceived of the project, supervised its design and coordination, and revised the manuscript. All authors have read and approved the final manuscript.
